# The *Caenorhabditis elegans sdhb-1(R244H)* Model Shows Characteristics of Human PPGL Tumor Cells

**DOI:** 10.3390/ijms262010185

**Published:** 2025-10-20

**Authors:** Fanni Ősz, Mahmood Akbar, Balázs Zoltán Zsidó, Csaba Hetényi, Tamás I. Orbán, Ábel Fóthi, Gábor M. Kovács, Alexandra Pintye, Attila Boda, Aamir Nazir, Zsolt Farkas, Krisztina Takács-Vellai

**Affiliations:** 1 Department of Biological Anthropology, Eötvös Lorand University, H-1117 Budapest, Hungary; oszfanni@student.elte.hu (F.Ő.); zsolt.farkas@ttk.elte.hu (Z.F.); 2 Academy of Scientific and Innovative Research (AcSIR), Ghaziabad 201002, India; mahmoodakbar27@gmail.com (M.A.); anazir@cdri.res.in (A.N.); 3 Division of Toxicology and Experimental Medicine, CSIR-Central Drug Research Institute, Lucknow 226031, India; 4 Pharmacoinformatics Unit, Department of Pharmacology and Pharmacotherapy, Medical School, University of Pécs, H-7624 Pécs, Hungary; zsido.balazs@pte.hu (B.Z.Z.); hetenyi.csaba@pte.hu (C.H.); 5 National Laboratory for Drug Research and Development, H-1117 Budapest, Hungary; 6 Gene Regulation Research Group, Institute of Molecular Life Sciences, HUN-REN Research Centre for Natural Sciences, H-1117 Budapest, Hungary; orban.tamas@ttk.hu (T.I.O.); fothi.abel@semmelweis.hu (Á.F.); 7 Department of Genetics, Cell- and Immunobiology, Semmelweis University, H-1089 Budapest, Hungary; 8 Department of Molecular Biology, Semmelweis University, H-1085 Budapest, Hungary; 9 Department of Plant Anatomy, Eötvös Lorand University, H-1117 Budapest, Hungary; gaborm.kovacs@ttk.elte.hu (G.M.K.); pintye.alexandra@atk.hun-ren.hu (A.P.); 10 Centre for Agricultural Research, Plant Protection Institute, HUN-REN, H-1116 Budapest, Hungary; 11 Department of Anatomy, Cell and Developmental Biology, Eötvös Lorand University, H-1117 Budapest, Hungary; attila.boda@ttk.elte.hu

**Keywords:** succinate dehydrogenase B, SDHB, hypoxia, ROS, mitophagy, SDH inhibitor, fluopyram, fungicide, dopaminergic neuronal damage

## Abstract

Pheochromocytomas and paragangliomas (PPGL) are classified as rare cancers but can be highly metastatic, particularly in individuals with inherited succinate dehydrogenase B (SDHB) mutations. As current therapies and the availability of *SDHB*-deficient animal models are both limited, we have previously constructed a nematode PPGL model, a transgenic worm carrying the R244H missense mutation equivalent to human R230H in the *sdhb-1* gene. In this study, we show that R244H mutants display characteristics of PPGL tumors, such as pseudohypoxia activation and the accumulation of reactive oxygen species. The latter can be the result of compromised antioxidant machinery, as R244H mutants have reduced levels of cytosolic and mitochondrial superoxide dismutase enzymes. In addition, the expression of mitophagy markers *pink-1* (PTEN-induced putative kinase) and *pdr-1* (E3 ubiquitin-protein ligase parkin) were downregulated in R244H mutants, suggesting impaired mitophagy and reflecting the crucial role of mitochondrial health in PPGL pathology. Treatments by the SDH inhibitor fluopyram revealed that the SDH complex carrying the R244H mutation in subunit B displayed residual SDH activity, which was also confirmed by our structural analyses. We also observed a link between dopaminergic neuronal health and SDHB-1.

## 1. Introduction

Pheochromocytomas (PHEO) and paragangliomas (PGL) are rare neuroendocrine tumors, which often have a delay in diagnosis [1]. PHEOs arising from the chromaffin cells of the adrenal medulla and PGLs derived from sympathetic and parasympathetic ganglia are now grouped together as PPGLs based on their similar genetic background and histological representation [2]. Although PPGLs are considered as rare cancers [3], they are also reported to have one of the highest degree of heritability out of all malignancies [4]. For example, significant numbers of PPGL patients harbor germ line succinate dehydrogenase subunit B (*SDHB*) mutations and suffer from a particularly aggressive form of malignant PPGL, for which there is no targeted therapy [5,6]. Furthermore, the absence of SDHB-mutant animal models [7] led us to create a novel *Caenorhabditis elegans* model carrying the equivalent of the clinically relevant R230H mutation of SDHB (R244H in the worm) [8]. Unlike *sdhb-1(-)* null mutant worms that display early larval arrest at the L2 stage, R244H worms reach adulthood but display slower development, sterility, and a shorter lifespan. R244H missense mutation results in a number of phenotypes including succinate accumulation, aberrant (Warburg’-like) glycolysis with increased expression of lactate dehydrogenase, decreased oxygen consumption, reduction in ATP production, and reduced mitochondrial number. Interestingly, both the Warburg effect [9] and increased lactate-dehydrogenase A (LDHA) expression have also been found in *SDHB*-deficient PPGL patients [10]. Our model not only recapitulates metabolic defects, but it is also drug-responsive, as treatment with the lactate-dehydrogenase inhibitor GSK2837808A [8] led to an increased L2/L3 larval arrest resembling the phenotypic appearance of *sdhb-1(-)* null mutant animals.

Herein we expand our studies, focusing on three areas: Firstly, we investigate the activation of pseudohypoxia and ROS accumulation, which are important hallmarks of PPGL tumors [11,12]. Secondly, with respect to some clinically relevant SDHB point mutants, structural predictions from crystallographic data suggest that the active site of the SDH enzyme is deformed but potentially still active [13]. However, there is no such data available about the human R230H SDHB mutation. Thus, we ask if the complex carrying the R244H mutant B subunit is assembled and if it is, does it retain SDH activity? Lastly, although a mutant copy of the ubiquitous *SDHB* gene is present in all cells of PPGL patients, it is observed that they develop tumors only in neuroendocrine tissues. This implies some unique functions of SDHB in these tissues. In order to obtain insight into this phenomenon we examined the function of SDHB-1 in the nervous system of the worm, focusing on dopaminergic neurons.

## 2. Results

### 2.1. Pseudohypoxia Is Activated Under Normoxic Conditions in sdhb-1(R244H) Mutants

**Figure 1:**
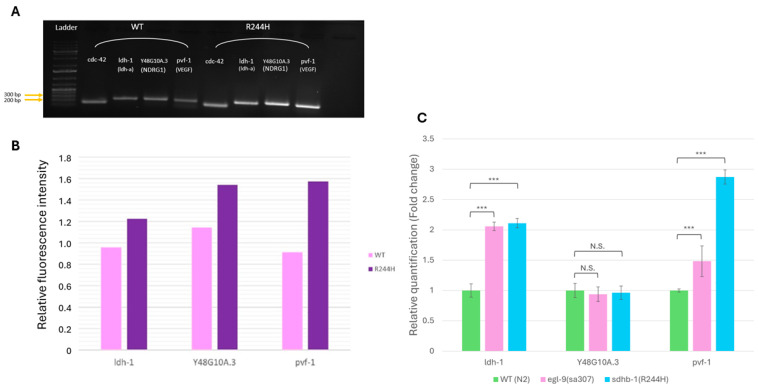
Analyzing pseudohypoxia in *sdhb-1(R244H)* mutants: analyzing the expression of HIF-1 target genes. (**A**,**B**) Semi-quantitative RT-PCR detected elevated mRNA levels of the *LDHA* homolog *ldh-1*, the *VEGFA* homolog *pvf-1*, and the *NDRG1* homolog *Y48G10A.3* in *sdhb-1(R244H)* mutants compared with WT worms (N2). (**C**) SYBR Green assay confirmed upregulation of *ldh-1* and *pvf-1* in *sdhb-1(R244H)* mutants. *egl-9(sa307)* mutants defective for prolyl hydroxylase function were used as a positive control. A representative measurement is shown; for the ASA reference gene, we used the ubiquitously expressed *cdc42* in all assays. For statistical analysis, Student’s *t*-test was applied, *** *p* < 0.001, N.S.: not significant, n = 3. Error bars represent S.D.

It is believed that in many cancer-linked SDH mutations mitochondrial succinate accumulates in the cytoplasm and activates HIF-1α (hypoxia-inducible factor alpha), which favors pseudohypoxia-driven tumorigenesis through mal-activation of the target genes [11]. Hence, we first analyzed pseudohypoxia in *sdhb-1(R244H)* mutants. *Caenorhabditis elegans* displays an evolutionarily conserved hypoxia-responsive system, the HIF-1α homolog hypoxia–inducible factor is encoded by the *hif-1* gene [14]. *C. elegans* HIF-1 is the single functional homolog of mammalian HIFα proteins, serving as the alpha subunit of the heterodimeric hypoxia–inducible factor (HIF) complex in the nematode. The N-terminus of HIF-1 containing the conserved bHLH and PAS domains shows high sequence similarity to the corresponding domains of human HIF-1α and HIF-2α [15]. Under normoxic conditions, *C. elegans* HIF-1 is regulated in a similar way to its human counterpart HIF-α: HIF-1 is hydroxylated by the prolyl hydroxylase EGL-9 and ubiquitinated by the VHL (von Hippel Lindau) homolog VHL-1, which leads to the proteasomal degradation of HIF-1. In contrast, if hypoxia occurs, HIF-1 remains stable and induces the transcription of the target genes [16]. Our previous transcriptomic and metabolic analyses showed that the HIF-1 target lactate-dehydrogenase *ldh-1* gene is only overexpressed in point mutants but not in *sdhb-1(gk165)* deletional mutants [8]. The null mutants were not examined further because we could not directly compare the point mutant adults with the L2 null mutant. Therefore, we concentrated on the R244H point mutants and measured the expression of several HIF-1 target genes by semi-quantitative RT-PCR in *sdhb-1(R244H)* mutant background compared with N2 adults (WT). *VEGFA* (*vascular endothelial growth factor A*), *LDHA* (*lactate-dehydrogenase A*) and *NDRG1* (*N-myc downstream regulated 1*) genes showed elevated expression in human cell line studies in response to HIF-1α activation [17]. We examined the expression of the worm homologs of the above HIF-1α targets and detected elevated mRNA levels of the *LDHA* homolog *ldh-1* (thus, confirming earlier data), the *VEGFA* homolog *pvf-1*, and the *NDRG1* homolog *Y48G10A.3* in *sdhb-1(R244H)* mutants compared with WT (N2) worms by semi-quantitative PCR (Figure 1A,B). Next, we performed real-time PCR (SYBR Green assay) using the same primers, and *egl-9(sa307)* mutants as a positive control. The deletion of EGL-9, a negative regulator of HIF-1, results in the constitutive activation of HIF-1, as described above. qPCR experiments confirmed the upregulation of *ldh-1* and *pvf-1*, in both *egl-9(sa307)* and *sdhb-1(R244H)* mutants as expected (Figure 1C).

**Figure 2:**
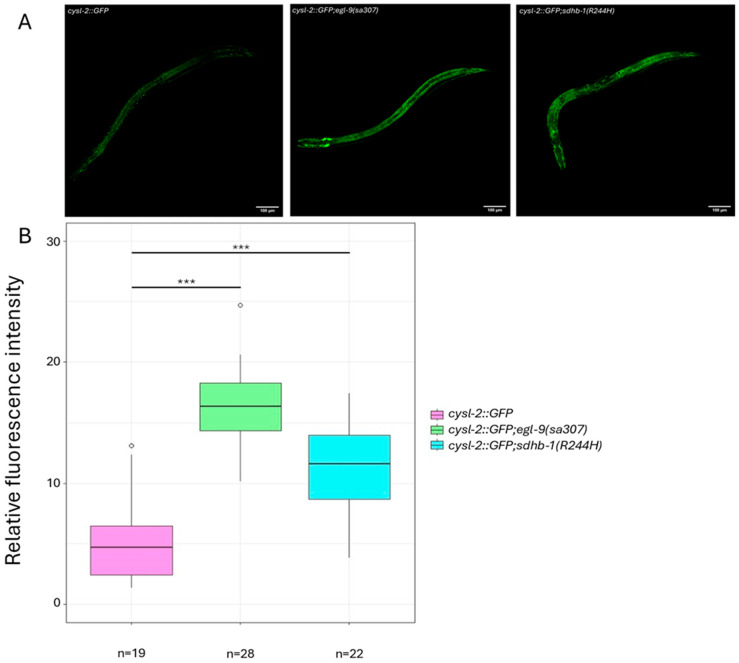
Pseudohypoxia is activated in *sdhb-1(R244H)* mutants. (**A**) *cysteine synthase-like 2 (cysl-2)* is a direct target of HIF-1; elevated *cysl-2::GFP* transgene expression is a marker of HIF-1 activity. HIF-1 becomes constitutively active in *cysl-2::GFP*; *egl-9(sa307)* mutants are defective for prolyl hydroxylase. *cysl-2::GFP* expression is also increased in *sdhb-1(R244H)* mutant background indicating activation of pseudohypoxia under normoxic conditions in these mutants. (**B**), Graphical representation of fluorescence intensity was quantified using Image J (for statistical analysis Mann–Whitney test was applied, *** *p* < 0.001).

In order to confirm the above qPCR data, we assayed HIF-1 activity by crossing the reporter *P_cysl-2_::GFP* into the *sdhb-1(R244H)* mutant background. The *cysl-2* (cysteine synthase-like) gene is one of the strongest regulated direct targets of HIF-1 [18]. As a positive control, we used *egl-9(sa307)* and *cysl-2::GFP* mutants, which are defective in prolyl hydroxylase function; thus, HIF-1 is constitutively active in these mutants and induces strong *cysl-2::GFP* expression (Figure 2A). We found that *cysl-2::GFP* is also activated in *sdhb-1(R244H)* mutants, similar to *egl-9(sa307)* mutants (Figure 2B).

Together, these results indicate that pseudohypoxia is activated under normoxic conditions in the R244H point mutant SDHB model due to succinate accumulation, just like in PPGL tumor cells.

### 2.2. Impact of sdhb-1 Null Mutation and R244H Point Mutation on Overall Oxidative Status

Loss-of-function mutations of the SDH complex not only disrupt the proper function of the TCA cycle but also impair the oxidative function of the mitochondrion by damaging the function of the electron transport chain (ETC). Defects of the ETC elevate reactive oxygen species (ROS), a classic byproduct in SDH deficiency, creating carcinogenic effects [19,20] because an excess of ROS damages the structure of macromolecules, e.g., DNA, proteins, and lipids of the cell membrane. Importantly, experiments conducted on model animals and cell cultures also show that mutations in SDH result in increased cellular ROS levels [21,22,23,24,25].

**Figure 3:**
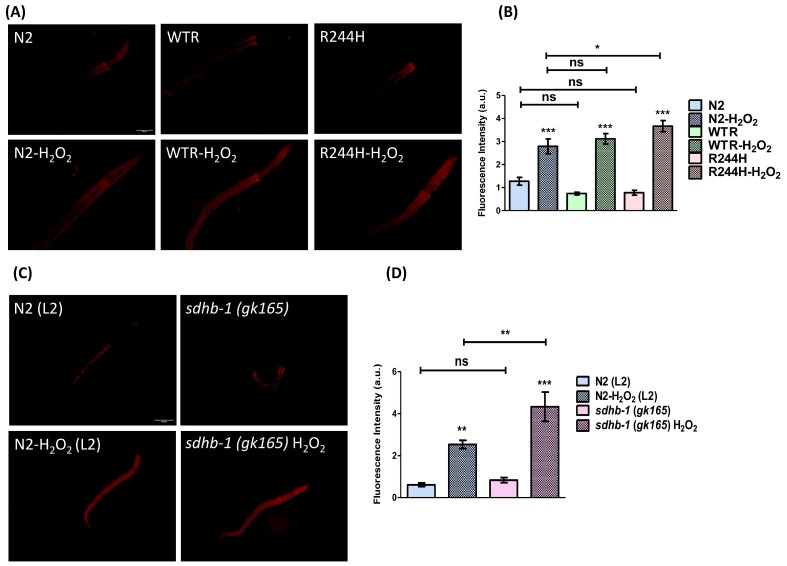
Effect of *sdhb-1* null mutation and Arg244His point mutation on ROS content detected by DHE staining. (**A**) Representative photomicrographs showing the level of ROS in N2, WTR, and R244H strains of *C. elegans* at the young adult stage (scale bar 50 μm). (**B**) Graphical representation of fluorescence intensity quantified using Image J software (for statistical analysis one way ANOVA (Tukey’s post hoc test was applied; n = 10; * *p* < 0.05, *** *p* < 0.001, and ns—non-significant; a.u.—arbitrary unit). (**C**) Representative photomicrographs showing the level of ROS in N2 and *sdhb-1(gk165)* mutant strains of *C. elegans* at L2 larval stage (scale bar 50 μm). (**D**) Graphical representation of fluorescence intensity quantified using Image J software (for statistical analysis one way ANOVA (Tukey’s post hoc test was applied; n = 10; ** *p* < 0.01, *** *p* < 0.001, and ns—non-significant; a.u.—arbitrary unit).

As the *sdhb-1* deletion and R244H point mutants have already been reported to show reduced mitochondrial and ATP content [8], in order to delineate the cause of this mitochondrial dysfunction, we examined the level of ROS by employing dihydroethidium (DHE) staining. This dye specifically stains superoxide ions, which are relatively short-lived. Its reaction with superoxide anions results in the formation of ethidium (E^+^) which binds to DNA and produces enhanced fluorescence [26,27]. The *sdhb-1* mutants (both null and point) and wild-type rescued (WTR, please see [8]) worms had no effect on the basal ROS level. However, the level of induced ROS in response to H_2_O_2_ treatment was found to be elevated in the cases of both *sdhb-1* null and R244H point mutants. In comparison with the wild type, R244H point mutants showed an increment by 0.8773 ± 0.3998 whereas in *sdhb-1* null mutants the level of induced ROS was increased by 1.790 ± 0.7266 (Figure 3).

**Figure 4:**
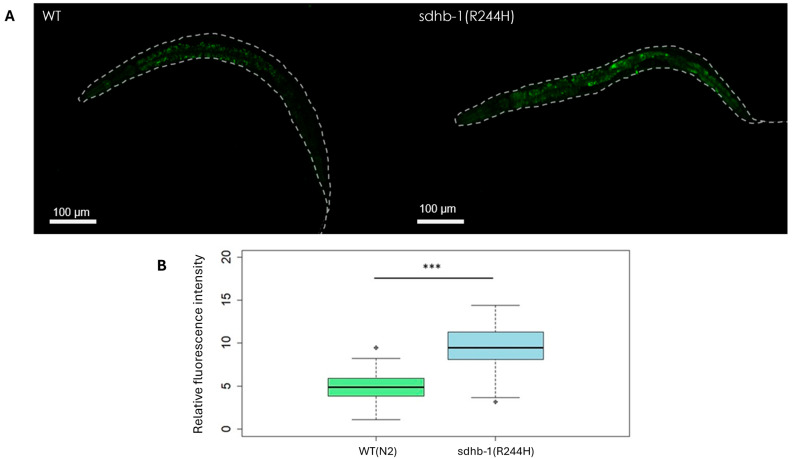
CellROX Green staining shows increased ROS levels in *sdhb-1(R244H)* mutants at the basal state. (**A**) In total, 37 wild-type and 37 mutant young adult *sdhb-1(R244H)* mutants were stained by CellROX Green and analyzed by epifluorescent microscopy (n = 37). (**B**) The boxplot shows a significant increase in fluorescence in R244H mutants compared with WT worms. Two-sample *t*-test was used to compare wild-type and mutant animals (***: *p* < 0.001).

The CellROX method uses a fluorogenic probe for measuring oxidative stress in live cells. This cell-permeant dye exhibits fluorescence upon oxidation by ROS and subsequent binding to mitochondrial DNA. As CellROX is able to detect different types of reactive oxygen species [28], not only superoxide anions, we decided to test R244H mutants by this method. As a result, similar to the data obtained in the PPGL cells, CellROX Green staining on R244H mutant worms also showed significantly increased ROS levels at the basal state (Figure 4).

### 2.3. Impact of sdhb-1 Null Mutation and R244H Point Mutation on Levels of Antioxidant Enzymes, Mitophagy and Apoptosis Markers

**Figure 5:**
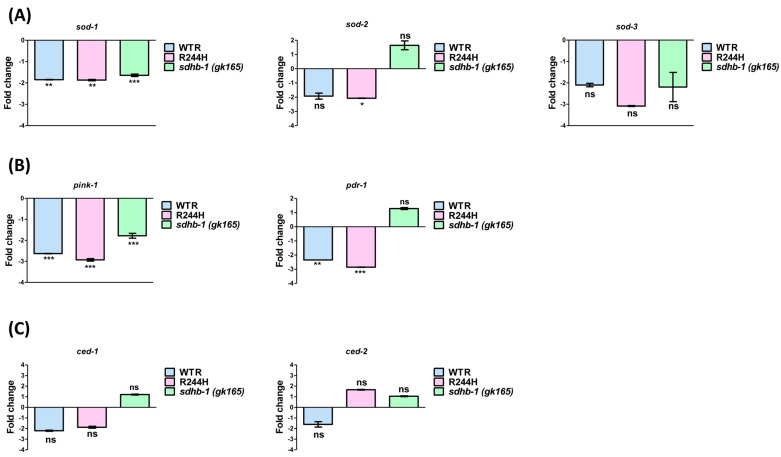
Effect of *sdhb-1* null mutation and Arg244His point mutation on antioxidant enzyme expression, mitophagy, and apoptosis markers. (**A**) Expression of *sod-1*, *sod-2,* and *sod-3* in the L2 larval stage of *sdhb-1(gk165)* mutant and young adult stage of WTR and R244H strains in comparison with stage-specific wild-type controls (one way ANOVA (Tukey’s post hoc test) n = 3; * *p* < 0.05, ** *p* < 0.01, *** *p* < 0.001, and ns—non-significant). (**B**) Expression of *pink-1* and *pdr-1* in the L2 larval stage of *sdhb-1(gk165)* mutant and young adult stage of WTR and R244H strains in comparison with stage-specific wild-type controls (** *p* < 0.01, *** *p* < 0.001, and ns—non-significant). (**C**) Expression of *ced-1* and *ced-2* in the L2 larval stage of *sdhb-1(gk165)* mutant and young adult stage of WTR and R244H strains in comparison with stage-specific wild-type controls (ns—non-significant). For statistical analysis, one way ANOVA (Tukey’s post hoc test) was applied; n = 3.

Antioxidant enzymes such as superoxide dismutases (SODs) compensate for excess ROS in healthy cells. To further investigate the inability of point and null mutants in subjugating oxidative insults, we examined the expression levels of *sod-1*, *sod-2,* and *sod-3* in mutant backgrounds. *sod-1* is a cytosolic SOD enzyme, whereas *sod-2* and *sod-3* are mitochondrial SOD enzymes [29]. In comparison with stage specific wild-type controls, the expression of *sod-2* and *sod-3* in *sdhb-1* null mutant was found to be insignificantly altered, but the expression of *sod-1* was significantly downregulated (1.64-fold). Moreover, in R244H point mutants, only *sod-1* and *sod-2* levels were found to be downregulated (1.87 and 2.07-fold, respectively), which was also observed in rescued worms (Figure 5A). Such downregulation of antioxidant enzymes might be responsible for the reduced tolerance of *sdhb-1* mutants against oxidative stress. As defects in the mitophagic process can contribute to the accumulation of dysfunctional mitochondria and oxidative imbalance, we also examined the expression levels of mitophagy markers, PTEN-induced putative kinase (PINK1/PINK-1), and the E3 ubiquitin-protein ligase parkin (PDR-1) under different mutant backgrounds. In order to induce mitophagy, PINK1/PINK-1 accumulates on the outer membrane of defective mitochondria and recruits parkin/PDR-1, which in turn forms polyubiquitin chains and promotes engulfment by autophagosomes [30]. The expression of both *pink-1* and *pdr-1* was found to be downregulated only in R244H point mutants (2.92 and 2.86 fold, respectively), suggesting impediment of mitophagic process and thereby accumulation of dysfunctional mitochondria and oxidative imbalance (Figure 5B). Since mitochondrial dysfunction leads to apoptosis induction, we also examined the expression of apoptosis markers *ced-1* and *ced-2*, which were not significantly altered under mutant backgrounds (Figure 5C).

### 2.4. Fluopyram Treatment Selectively Affects the Development of sdhb-1(R244H) Mutants

In our earlier work [8], we showed that the human SDHB R230H mutation causes a conformational change in the B subunit of the SDH complex, which also damages the A–B interface. These structural studies led to the conclusion that the complex carrying the R230H mutation was either inactive or had an altered SDH activity.

**Figure 6:**
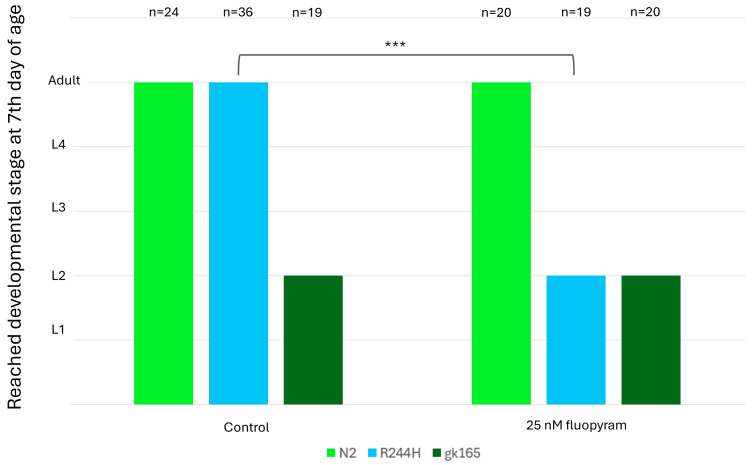
The 25 nM fluopyram treatment selectively affects the development of *sdhb-1(R244H)* mutants. Development of wild-type (N2) animals (light green) and *sdhb-1(gk165)* null mutants (dark green) are not influenced by 25 nM fluopyram. R244H mutants (blue), which reach adult stage on the control plates, are arrested in the L2 larval stage (e.g., null mutant phenotype) in response to fluopyram treatment at day 7. Statistical analysis was performed by the Wilcoxon test, *** *p* < 0.001.

To see if the R230H equivalent of the R244H mutation in the worm results in residual SDH activity, we applied SDHB-inhibitor treatment on *sdhb-1* mutants. Fluopyram is a worm-selective fungicide, which blocks the ubiquinone binding pocket of SDH at the interface of SDHB and C and D subunits [31]. We treated wild-type and *sdhb-1* null and point mutant worms with fluopyram, which caused complete and partial embryonic lethality of all the strains examined at 2.5 μM and 250 nM concentrations, respectively, (also observed in [31]); 25 nM fluopyram treatment did not affect the viability and development of N2 animals and *sdhb-1(gk165)* null mutants. However, R244H mutants, which normally reach adulthood, showed L2 larval lethality, e.g., null mutant phenotype, in response to 25 nM fluopyram (Figure 6). These data suggest that R244H mutants display a residual SDH activity.

### 2.5. Binding Mode of Fluopyram to the Wild-Type and Mutant SDH Complex

**Figure 7:**
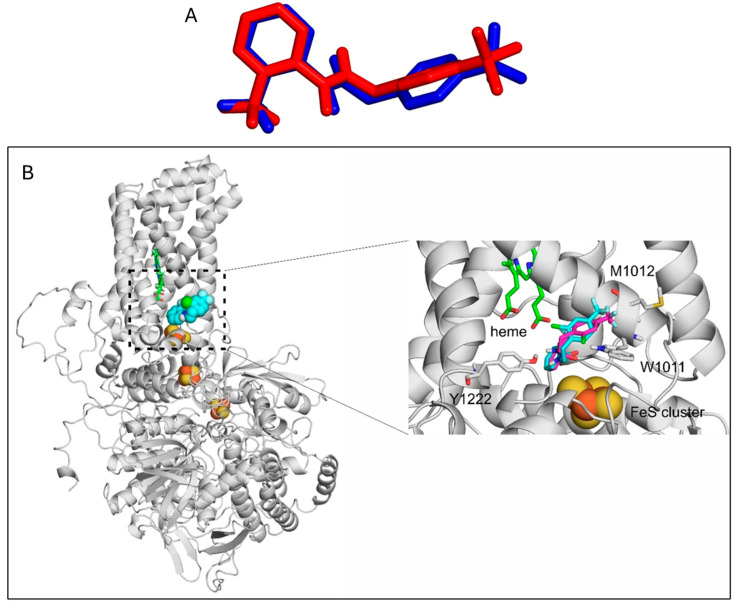
(**A**) Re-docking of flutolanil to PDB:4ytp. The red molecule is the crystallographic and the blue is the docked binding mode. (**B**) The homology model of the *Caenorhabditis elegans* mitochondrial complex II in complex with fluopyram. On the left, the whole protein complex is shown with a gray cartoon, the heme is represented as green sticks, the FeS clusters as yellow and orange spheres, and the bound fluopyram as blue spheres. On the right, a close-up of the fluopyram binding site is shown, and the interacting amino acids are shown as gray sticks. The highlighted amino acids are identical to C chain W35, M41, and D chain Y91 in the PDB:4ytp structure. The binding mode of fluopyram to the wild-type protein is shown as teal sticks, and to the mutant protein as purple sticks.

As experimental validation, the re-docking of flutolanil to the *Sus scrofa* heart mitochondrial complex II (SDH complex) was successful, the RMSD of the docked binding mode compared with the experimental binding mode was 1.6 Å (Figure 7A). The same methodology was applied for docking fluopyram to the *Caenorhabditis elegans* mitochondrial complex II.

Fluopyram binds similarly to the *Caenorhabditis elegans* mitochondrial complex II as flutolanil does to the *Sus scrofa* heart mitochondrial complex II, to the interface of the B and C chains. Both the proteins and the ligands are structurally similar, which explains the similar binding mode. The R244H mutation at the A/B chain interface did not affect ligand binding at the B/C chain significantly. The binding mode of fluopyram to the wild type and mutant proteins are almost identical. The difference in the binding free energy of flutolanil binding to *Sus scrofa* heart mitochondrial complex II and fluopyram binding to the wild type and mutant *Caenorhabditis elegans* mitochondrial complex II is smaller than 0.2 kcal/mol, which is not a significant difference. The amide N of fluopyram interacts with the hydroxyl group of Y1222 (Figure 7B), the pyridine ring interacts with the rings of W1011, and the fluor atoms form hydrophobic interactions with M1012. Thus, the R244H mutant *Caenorhabditis elegans* mitochondrial complex II can bind to fluopyram, explaining its experimental inhibitory effect, consistent with our proposed residual enzyme activity.

### 2.6. Expression Pattern of SDHB-1 and DAT-1 Throughout Different Stages of Worm Development

**Figure 8:**
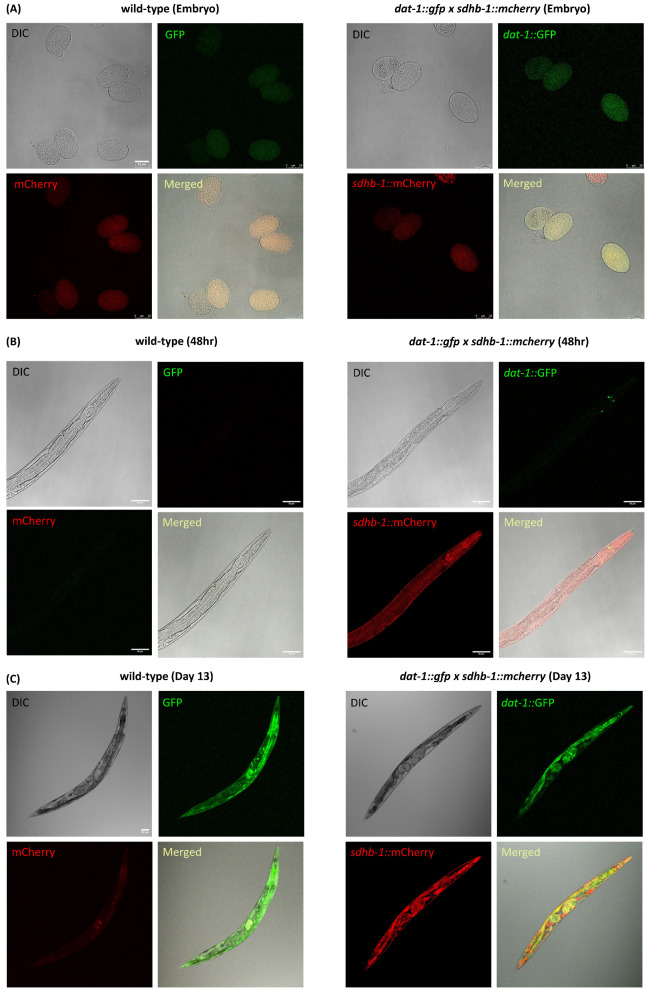
SDHB-1 is ubiquitously expressed along with DAT-1 throughout worm development. (**A**) Confocal microscopy images of SDHB-1 and DAT-1 expression at the embryo stage of *dat-1::gfp* x *sdhb-1::mCherry* worm development. Scale bar: 25 µm. (**B**) Confocal microscopy images of SDHB-1 and DAT-1 expression at the 48 h stage of *dat-1::gfp* x *sdhb-1::mCherry* worm development. Scale bar: 50 µm. (**C**) Confocal microscopy images of SDHB-1 and DAT-1 expressions at the day 13 stage of *dat-1::gfp* x *sdhb-1::mCherry* worm development. n = 3.

Some patients with SDHB-related PPGL tumors have an excess level of catecholamine secretion; however, the exact role of this excess secretion in tumor progression is not entirely known. In *Caenorhabditis elegans* four biogenic amines are present: dopamine, serotonin, octopamine, and tyramine. Dopamine—whose receptors and downstream signaling pathways are highly similar to their mammalian counterparts—is an important neurotransmitter responsible for changes in locomotion, behavior, and learning upon changes in the environment [32]. As a first approach to examine the link between dopamine production/secretion and SDHB-1 function in the worm, we attempted to identify dopaminergic neurons, which express SDHB-1 as well. To achieve this goal, we first generated a transgenic strain carrying a p_sdhb-1_::SDHB-1::mCherry construct and crossed this strain with a DAT-1::GFP reporter, which labels dopaminergic neurons specifically (DAT-1 possess dopamine–sodium symporter activity; [33]). We studied the expression of SDHB-1::mCherry and DAT-1::GFP throughout different stages of worm development, i.e., embryos, 48 h, and day 13, by employing confocal microscopy. In comparison with N2, the expression of both DAT-1 and SDHB-1 was distinctly visible and persisted throughout all the studied developmental stages. The expression of DAT-1 remained confined in the dopaminergic neurons, whereas SDHB-1 expression was mainly observed in pharynx, hypodermis, and intestine (Figure 8). At day 13, both N2 and *dat-1::gfp* x *sdhb-1::mCherry* strains show intense green signals in the intestine due to accumulation of “age-pigment” or lipofuscin. However, the expression of DAT-1 is distinctly visible in the cephalic region of double transgenic worms.

### 2.7. Correlating Mitochondrial Dysfunction and Dopaminergic Neurodegeneration in the Context of B Subunit of Succinate Dehydrogenase (SDHB)

**Figure 9:**
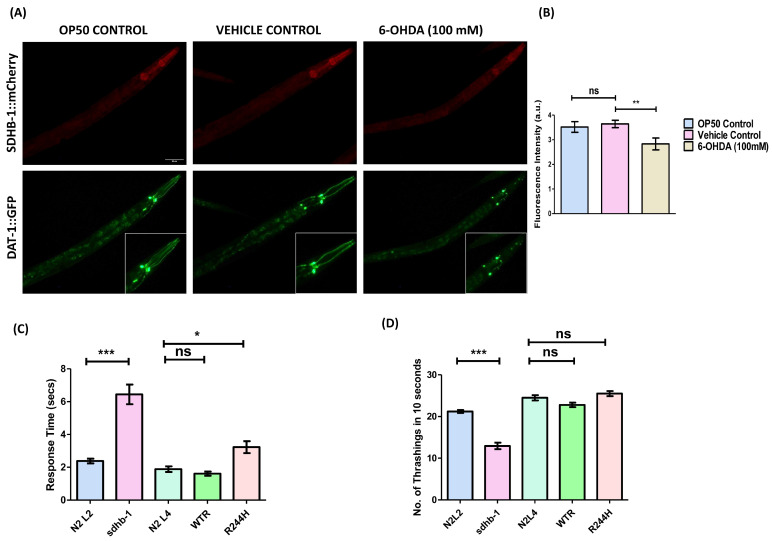
Correlation between SDHB-1 activity and dopaminergic neuronal health. (**A**) Representative fluorescent images showing the expression of SDHB-1::mCherry and DAT-1::GFP in day 3 worms upon transient exposure to 6-OHDA (100 mM) during the early larval stage; the white box shows the digitally magnified image of the region under investigation; scale bar: 50 µm. (**B**) Quantification of fluorescence intensity pertaining to SDHB-1::mCherry expression (for statistical analysis two-tailed Student’s *t*-test was applied; n = 20 worms per condition; ns: non-significant and ** *p* < 0.01; a.u.—arbitrary unit). (**C**) Graphical representation of response time against 1-nonanol, a known repellent used to assess dopaminergic health (two-tailed Student’s *t*-test for statistical analysis; n = 10 worms per condition; ns: non-significant, * *p* < 0.05, and *** *p* < 0.001). (**D**) Graphical representation of the number of thrashes made by the worm in 10 s (one way ANOVA (Tukey’s post hoc test) n = 20 worms per condition; ns: non-significant and *** *p* < 0.001).

In order to study the effect of dopaminergic neuronal damage on SDHB-1 expression, we subjected *dat-1::gfp* x *sdhb-1::mCherry* worms to 6-hydroxydopamine (6-OHDA) damage during the early larval stage. 6-OHDA is a neurotoxin that selectively targets dopaminergic neurons by inducing mitochondrial dysfunction and oxidative damage [34]. The exposure to 100 mM 6-OHDA was given at the L3 larval stage, transiently, and was sufficient to cause substantial dopaminergic neuronal damage till the young adult stage (Figure 9A,B). The loss of dopaminergic neurons at the young adult stage was found to significantly lower the expression of SDHB-1 (0.8115 ± 0.2848) as compared with the vehicle control. SDHB-1 has been reported to play an important role in mitochondrial electron transport chain and tricarboxylic acid cycle. Its downregulation upon selective oxidative damage to dopaminergic neurons supports a mechanistic link between mitochondrial dysfunction and dopaminergic neuronal dysfunction.

To further explore the functional consequences, we examined the status of dopamine related neuro-behaviors in *sdhb-1* mutants—both null and point mutants (Figure 9C,D). In these mutants, we performed thrashing and a 1-nonanol aversion assay to assess the effect on established dopamine-associated endpoints including locomotion and olfaction, respectively, [35]. The null mutant exhibited significantly impaired dopaminergic functions, evident by a slower thrashing rate and delayed response to the repellent, 1-nonanol. In comparison with age-specific wild-type control, the thrashing rate of *sdhb-1* null mutant declined by 8.250 ± 0.8678 thrashes, and the response time was increased by 4.062 ± 0.6190 s. The R244H point mutant only showed olfactory impairment as the response time was slightly increased by 1.345 ± 0.4042 s in comparison with the control, indicating partial retention of SDH activity.

## 3. Discussion

As there is no definitive therapy for PPGL, model systems are important in both understanding the pathophysiology of the disease and the screening of drug candidates. Since availability of *SDHB*-deficient animal models is limited, we have previously constructed a transgenic worm carrying the R244H missense mutation in the *sdhb-1* gene, which is equivalent to human R230H [8]. In this study, we performed an extensive characterization of the model, firstly by focusing on several hallmarks of PPGL tumor cells, such as the activation of pseudohypoxia and ROS accumulation.

*Caenorhabditis elegans* displays an evolutionarily conserved hypoxia-responsive system. We examined the expression of several conserved HIF-1 target genes in R244H mutants by semi-quantitative and quantitative PCR and found elevated expressions of the *VEGFA* homolog *pvf-1* and the *LDHA* homolog *ldh-1* with both methods, while the *NDRG1* homolog *Y48G10A.3* gene showed elevated mRNA level by semi-quantitative PCR. A seminal paper has recently been published about identifying conserved HIF-1 target genes in *C. elegans* [36]. Some HIF-1 targets described by Vora et al. such as *ldh-1*, *mdh-1* (malate dehydrogenase), and PEP carboxykinase *pck-1* were also selectively activated in the R244H mutants ([8] and this study). We also confirmed pseudohypoxia activation by crossing a reporter, *cysl-2::GFP*, into R244H mutants. *cysl-2* is one of the strongest regulated direct targets of HIF-1 [18]. We found that *cysl-2::GFP* expression was induced in the *sdhb-1(R244H)* background, similar to the *cysl-2::GFP* and *egl-9(sa307)* mutants, which lack prolyl hydroxylase function. *cysl-2::GFP* and *sdhb-1(R244H)* double mutants could be used as a tool to screen HIF inhibitors [37].

Furthermore, our results show that SDHB mutations can significantly influence the overall oxidative status in *C. elegans*. We measured ROS levels in *sdhb-1(-)* null and R244H point mutants by two different methods. DHE staining, which primarily stains superoxide anions, resulted in no increase in the basal ROS state in any *sdhb-1* mutant background. However, when R244H point mutants were tested by the CellROX Green method, which detects different types of reactive oxygen species, we found an increase in the basal ROS state, similar to data obtained in PPGL cells. Upon oxidative insult, both R244H point mutants and *sdhb-1* null mutants presented a successive increment in ROS content, pointing towards their inability to tolerate oxidative stress as compared with the control. Upon investigation, this inability to subjugate oxidative insults was found to be a result of compromised antioxidant machinery. Both mutants showed marked reduction in the level of *sod-1* enzyme, which is a cytosolic SOD enzyme. However, only R244H point mutants had a reduced level of a mitochondrial SOD enzyme, i.e., *sod-2*, reflecting imbalance in oxidative homeostasis at both cytosolic and mitochondrial levels. Additionally, based on our gene expression data, the point mutant also had compromised mitophagy, which further explained their susceptibility towards oxidative insults. Our previous assessment of mitochondrial health in null mutants has also revealed compromised mitochondrial function and bioenergetics [8]. These findings also suggest that both SDHB mutations alter the overall oxidative homeostasis and have defective mitochondrial physiology that contributes towards PPGL pathophysiology.

The near-ubiquitous use of fungicides with succinate dehydrogenase inhibitor (SDHi) activity in agriculture raises serious concerns, as SDHi can inhibit SDH activity in non-target species [38], for example in worms as well. In our work, the fungicide fluopyram is a worm-selective SDHi that inhibited R244H mutants selectively at 25 nM concentration, suggesting that these mutants display a residual SDH activity. Binding of fluopyram to the wild-type and R244H mutant *C. elegans* SDH complexes were also simulated in silico. We found that the binding mode of fluopyram to the wild-type and R244H mutant complexes were almost identical, consistent with the notion that the mutant SDH complex is assembled and explaining the experimental inhibitory effect of fluopyram on the residual enzymatic activity of R244H mutants.

Our investigations have also generated key insights into the behavior of SDHB and DAT-1 across various developmental stages, including the impact of dopaminergic neuronal damage on SDHB-1 expression, and the effects of *sdhb-1* mutations on overall dopaminergic functioning. The link observed between dopaminergic neuronal damage and SDHB-1 suggests an interesting connection between mitochondrial function and decline in neurotransmitter efficiency.

Co-existence of Parkinson’s disease (PD) with pheochromocytoma is rare as only five such cases have been described till date [39]. This concurrence of Parkinson’s disease and the medications used by its patients is known to interfere with the standard biochemical tests of pheochromocytoma, thus necessitating caution while diagnosis [40]. In pheochromocytoma, activation of the HIF-signaling pathway is deemed responsible, whereas in Parkinson’s disease and other neurodegenerative diseases it is considered as protective [41]. Intriguingly, in one of the reported cases of this co-existence, the levels of HIF-1α in the tumor were markedly diminished, which are found to be elevated in pheochromocytoma patients without Parkinson’s disease [39]. This signifies that the role of pseudohypoxia in the etiology of both the diseases warrants further attention. However, the mitochondrial dysfunction and oxidative imbalance observed in both diseases can explain such rare events more conclusively. Such cases provide opportunities to understand the different pathological outcomes of the same underlying cellular and molecular events. In our study, we have also examined such correlations between the two diseases in the context of mitochondrial dysfunction and oxidative imbalance. The subtle damage of dopaminergic neurons by oxidative assaults, exerted by 6-OHDA, and its systemic manifestations on SDHB-1 levels, suggest the existence of a yet unidentified mechanism that can transmit oxidative insults to and from in such cases. The existence of such a bidirectional mechanism is also substantiated by the compromised dopamine-related neuro-behaviors observed in the *sdhb-1* null mutant. Moreover, mutations in succinate dehydrogenase have been implicated in the onset of neurodegenerative disorders including Parkinson’s disease, by its virtue to regulate oxidative stress, lipid homeostasis and neuronal excitotoxicity [42]. This implies the existence of shared molecular underpinnings by both diseases, which are more comprehensible in the context of oxidative imbalance and mitochondrial dysfunction than the pseudohypoxia pathway. Our model provides an opportunity to study such concurrences and delineate the molecular events, which we will be carrying out in our future work.

The nematode is gaining momentum as a screening tool in drug discovery, as it combines genetic amenability, low cost, and culture conditions compatible with high throughput screening [43]. The FDA considers the worm as an alternative model in toxicity testing (https://www.fda.gov/food/toxicology-research/c-elegans-model-toxicity-testing (accessed on 30 September 2022)). This study shows that our *C*. *elegans* model can serve as a pharmacological model of succinate dehydrogenase loss disorders, as it is druggable and shows several characteristics of PPGL tumors. Our data also point to a potential problem with SDH-directed fungicides should our findings in *sdhb-1* mutant worms translate into humans with inherited *SDHB* variants. Such individuals may be uniquely susceptible to low concentrations of fungicides targeting *SDHB*, whereas their wild-type compatriots remain unaffected.

## 4. Materials and Methods

### 4.1. Caenorhabditis elegans Strains

*C aenorhabditis elegans* strains were maintained under standard conditions [44]. The following strains were used: wild type (WT), Bristol N2; VC294, {*sdhb-1(gk165)/ mIn1 (mIs14 [myo-2::gfp; pes-10::gfp]) dpy-10(e128)] II.*}; R244H, *sdhb-1(gk165)/ mIn1 [mIs14 [myo-2::gfp; pes-10::gfp] dpy-10(e128)] II.*; *unc-119(ed3) III.(?)*; *pNU636 (Psdhb-1_sdhb-1(G731A)_UTRsdhb-1*; *unc-119(+)) X.*; WTR, *sdhb-1(gk165)/ sdhb-1(gk165) II.*; *unc-119(ed3) III.(?)*; *pNU637 (Psdhb-1_sdhb-1(genomic wt)_UTRsdhb-1*; *unc-119(+)) X*.; TBV03, *sdhb-1*::mCherry, and the strain obtained from crossing TBV03 with TG2401, {dat-1(ok157) III; vtIs1 V; tsp-17(gt1681) X}.

### 4.2. Oxidative Stress Measurement

For ROS estimation by dihydroethidium (DHE) dye, Invitrogen D1168, worms were harvested and washed to remove adherent bacteria. The worms were then incubated in 5 μM DHE dye solution for 45 min on a rotor shaker (iRoll PR35, Accumax, New Delhi, India). For positive control group, 20 mM H_2_O_2_ was added along with DHE. After incubation, worms were washed with 1X PBS followed by M9 buffer (3 g KH_2_PO_4_, 6 g Na_2_HPO_4_, 5 g NaCl, 1 mL 1 M MgSO_4_ in 1 L H_2_O) [45]. Slide preparation for fluorescence image acquisition and statistical analysis of the obtained data was performed as mentioned earlier. CellROX Green was performed as described in [46].

### 4.3. Image Acquisition

For imaging, synchronous population of worms or embryos was first harvested and then washed thrice by M9 buffer to remove adhering bacteria. In the case of worms, immobilization was performed by using 100 mM sodium azide solution (Sigma, Cat. No. 71289 St. Louis, MO, USA). Immobilized worms were mounted on a clean glass slide, and a coverslip was placed over it. Fluorescence images were acquired by using Carl Zeiss Axio Imager M2 or M3 (Oberkochen, Baden-Württemberg, Germany) fluorescence microscope and for confocal microscopy, we used Leica SP8 confocal microscope, (Wetzlar, Hesse, Germany). The fluorescence intensity was quantified using ImageJ v.1.53t software (ImageJ, National Institute of Health, Bethesda, MD, USA).

### 4.4. Thrashing Assay

To evaluate the functional integrity of dopaminergic neural circuitry, a thrashing assay was conducted in *C. elegans* with appropriate age-specific controls. Briefly, a drop of M9 buffer was placed over NGM plate containing the worms. After a period of acclimatization (30 s), a video was recorded for another 30 s using Leica LAS EZ software (Version 3.4.0). The thrashing rate of each worm was manually counted for 10 s by snipping and slowing down the video file. For each group, 10 worms were evaluated, and the experiment was conducted in two separate biological repeats.

### 4.5. 1-Nonanol Aversion Assay

1-nonanol aversion assay is a chemotactic assay used to assess the olfactory response of *C. elegans* against a known repellent 1-nonanol [35]. This is used as a functional read-out for the health of dopaminergic neurons. Briefly, a metallic wire dipped in 1-nonanol is brought near to the anterior end of worm, and the time taken by each worm to respond is noted. For each group, 10 worms were evaluated, and the experiment was conducted in two separate biological repeats.

### 4.6. qRT-PCR and Semi-qPCR Experiments

The expression patterns of *hif-1* target genes were determined using semi-quantitative and quantitative real-time PCR (qRT-PCR) by relative quantification. Total mRNA content was extracted from 2000 animals per strain with MACHEREY-NAGEL NucleoSpin^®^ RNA RNA Isolation kit (Düren, North Rhine-Westphalia, Germany). For both semi-quantitative and quantitative real-time PCR (qRT-PCR) experiments, 500 ng of total RNA was reverse-transcribed using a High-Capacity RNA-to-cDNA kit (Applied Biosystems High-Capacity cDNA Reverse Transcription Kit (#2705108), Waltham, MA, USA) according to the manufacturer’s instructions. cDNA was diluted 10x. All measurements were performed in triplicate. Relative expression levels were calculated by the deltadeltaCT (ddCT) method using *cdc-42* as endogenous control [47]. Fold-change values were calculated from 2−ddCT.

The following primer pairs have been used: *cdc-42:* 5′-GACAATTACGCCGTCACAGTAATG-3′, 5′-TGAAGCTGGAGCAACCACG-3′ *pvf-1:* 5′-GATTCCACAACTGTCCGACG-3′, 5′-GCAATCAAAGCACGAACACG-3′ *ldh-1*: 5′-AGTCTTCGCGGAAATTGCAG-3′, 5′-GCCAGCGGTGATAGAGTAGT-3′ *NDRG1*: 5′-TTTACACGTGACATGCAGCC-3′, 5′-GTCCCGGAGCATTCACATTG-3′

SYBR Green-based (Applied Biosystems™ Power SYBR™ Green Master Mix (#2012607)

RT-qPCR reactions were set up in QuantStudio™ 3 Real-Time PCR System (Applied Biosystems).

Amplification data were analyzed using QuantStudio Design and Analysis Online Software (ThermoFisher Scientific, Waltham, MA, USA).

Semi-qPCR fluorescence intensities were calculated by the use of ImageJ v. 1.54g software.

### 4.7. Single Worm qRT-PCR Experiments

For RNA isolation from a single worm of N2, WTR, and R244H strains, a young adult worm was picked and washed in DEPC-treated water. After washing, the worm was suspended in 1 μL of lysis buffer (Tris 5 mM pH 8.6; EDTA 0.25 mM; 0.5% Triton X-100; 0.5% Tween 20 and 1 mg/mL of proteinase K) placed on the cap of a PCR tube. For the *sdhb-1* null mutant strain, L2 stage worms were subjected to RNA isolation with N2 L2 stage worms as reference. The PCR tube was briefly centrifuged to settle the worm suspension down and then incubated at 65 °C for 11 min on Sure Cycler 8800 PCR (Agilent Technologies, Santa Clara, CA, USA). To inactivate proteinase K, the tube was heated at 85 °C for 1 min and then immediately stored at 4 °C. For longer storage, isolated RNA was kept at −80° C [48]. Synthesis of cDNA was performed in accordance with the Verso cDNA synthesis kit (Thermo Scientic, Cat. No. AB-1453/B), and the cDNA synthesis protocol was run on Sure Cycler 8800 PCR (Agilent Technologies).

The genes of interest were quantitatively assessed using a TB Green^®^ Premix Ex Taq^TM^ (TaKaRa, Cat. No. RR420A, Bio, Otsu, Japan). As per the manufacturer’s instructions, the amount of cDNA in each well of 96 well Hi-Plate for Real Time (TaKaRa, Cat. No. NJ400, Bio, Otsu, Japan) was calculated to be 100 ng. Each sample was amplified in duplicates by using CFX96 Touch Real-Time PCR Detection System (Bio-Rad, Berkley, CA, USA). Each PCR cycle consisted of (i) 1 cycle of pre-incubation: 95 °C for 30 s and (ii) 40 cycles for amplification: 95 °C for 5 s, 55 °C for 30 s, and 60 °C for 35 s. The Ct values obtained were analyzed to calculate the relative fold change for each gene, with respect to actin as an internal reference. The primers used for different genes are listed in Table 1.

### 4.8. 6-OHDA Treatment

Stock solutions of 6-hydroxydopamine (500 mM) and ascorbic acid (200 mM) were prepared in double distilled water. For treatment, L3 larval stage worms of the *dat-1:gfp* x *sdhb-1:mCherry* strain were suspended in 200 μL of M9 buffer, which contained 100 mM 6-OHDA and 20 mM ascorbic acid. After treatment for 1 h at 24 °C with regular shaking, 6OHDA was rendered inactive by the addition of the M9 buffer, and the worms were washed and added to the OP50 seeded NGM plates to allow their growth till the young adult stage. Treatment with 20 mM ascorbic acid alone was considered as the vehicle control.

### 4.9. Residual SDH Activity

Fluopyram was added to NGM plates; animals were incubated on drug-containing and control plates for 7 days at 16 °C. Next, developmental stages reached by treated and non-treated worms were scored.

### 4.10. Modeling Studies

#### 4.10.1. Ligand Preparation

Fluopyram (*N*-[2-[3-chloro-5-(trifluoromethyl)pyridin-2-yl]ethyl]-2-(trifluoromethyl)benzamide) was built and energy minimized in Maestro [49].

Flutolanil (*N*-[(4-tert-butylphenyl)methyl]-2-(trifluoromethyl)benzamide) was acquired from the Protein Data Bank (PDB, [50]) under the code PDB:4ytp [51]. The ligands were equipped with partial charges and converted to pdbqt format in AutoDock Tools 4.2.6 [52].

#### 4.10.2. Target Preparation

The atomic coordinates of the *Sus scrofa* heart mitochondrial complex II in complex with flutonalil was obtained from the Protein Data Bank [50] with PDB code 4ytp [51]. The amino acid sequence of the *Caenorhabditis elegans* mitochondrial complex II subunit A (Q09508), B (Q09545), C (P41956), and D (O62215) were obtained from UniProt [53]. The atomic coordinates of the *Caenorhabditis elegans* mitochondrial complex II were built with AlphaFold 3 on the AlphaFold Server [54]. Atomic coordinates of the heme and FeS clusters were superimposed from PDB:4ytp. The partial charges of the heme were used from [55] as described in [56]. Partial charges of the FeS cluster close to the ligand binding site were calculated with MOPAC [57] with a PM7 parametrization [58]. The R244H mutant of the *Caenorhabditis elegans* mitochondrial complex II was built in PyMol 3.1 [59] and energy minimized with a steepest descent and conjugate gradient protocol in GROMACS 2020.1-1 [60]. Briefly, the protein was placed into a box filled with TIP3P [61] water molecules, and counter-ions were added to the system to neutralize charges. The AMBER [62] force field was used to build the topology of the protein. During the minimizations, exit tolerance levels were set to 500 and 200 kJ × mol^–1^ × nm^–1^, and the maximum step sizes were set to 0.5 and 0.75 nm for the steepest descent and conjugate gradient steps, respectively. Gasteiger-Marsili [63] partial charges were added to proteins in AutoDock Tools [52] to prepare for docking.

#### 4.10.3. Docking

The docking calculations were performed in AutoDock [52]. First, re-docking of flutonalil to PDB:4ytp was performed, then docking of fluopyram to the homology model of the wild type and mutant *Caenorhabditis elegans* mitochondrial complex II were performed with the same methodology. That is, the grid box was set to coordinates −20.33, −23.77, and 62.04 (the average coordinates of the bound flutonalil), with 30 × 30 × 30 grid points and 0.375 grid spacing. A Lamarckian genetic algorithm was applied with pseudo-Solis and Wets local search and a population size of 250 for 10 docking runs [64,65,66]. The generated ligand-binding modes were clustered and ranked according to their calculated free energy of binding; a lower rank corresponds to a more favorable binding energy. In the re-docking, a root mean square deviation (RMSD) was calculated between the heavy atoms of the experimental and the docked binding modes.

## 5. Conclusions

Our results in this paper show that several evolutionarily conserved HIF-1(hypoxia-inducible factor-1) target genes are induced in *sdhb-1(R244H)* mutants, moreover a reporter carrying the promoter of cysteine synthase like 2 (*cysl-2*), which is a strongly regulated HIF-1 target gene, is also activated in *sdhb-1* point mutants, indicating activation of hypoxia under normoxic conditions. Upon oxidative insult, reactive oxygen species (ROS) are accumulated in the *sdhb-1(R244H)* mutants. We found a decreased expression of cytosolic and mitochondrial superoxide dismutases, SOD-1 and SOD-2, respectively, in R244H mutants, which explains the imbalance in oxidative homeostasis. Expression levels of *pink-1* (PTEN-induced putative kinase) and *pdr-1* (E3 ubiquitin-protein ligase parkin) are downregulated in R244H mutants, suggesting impaired mitophagy. Our experimental results using the SDH inhibitor fluopyram and structural analyses show that the SDH complex carrying the R244H mutation in the B subunit displays residual SDH activity. In addition, our studies revealed a link between SDHB-1 and dopaminergic neuronal health.

Together, we propose our *C. elegans* model as a pharmacological model of succinate dehydrogenase loss disorders, as it is druggable and shows several characteristics of PPGL tumors.

## Figures and Tables

**Table 1:** Primers used for single worm qRT-PCR experiments.

Gene Name	Primer Sequences
*act-1*	Forward Primer: TTACTCTTTCACCACCACCGCTGA Reverse Primer: TCGTTTCCGACGGTGATGACTTGT
*od-1*	Forward Primer: CCAGGCAGTTATTGAAGGAGAA Reverse Primer: TGTGGACCGGCAGAAATG
*sod-2*	Forward Primer: GAGGCGGTCTCCAAAGGAAA Reverse Primer: CCAGAGATCCGAAGTCGCTC
*sod-3*	Forward Primer: CTCCAAGCACACTCTCCCAG Reverse Primer: TCCCTTTCGAAACAGCCTCG
*pink-1*	Forward Primer: AGTCGTCTGGACAAAGTGATG Reverse Primer: TTGCTCGAAGTTGTCGTTCT
*pdr-1*	Forward Primer: CAGACGTCGTACAGCGAATAC Reverse Primer: TCATAGGGCTCCCAGAAGAA

## Data Availability

The original contributions presented in this study are included in the article. Further inquiries can be directed at the corresponding author.

## Abbreviations

The following abbreviations are used in this manuscript:

DAT-1	dopamine transporter
DHE	dihydroethidium
ETC	electron transport chain
HIF	hypoxia-inducible factor
LDH	lactate dehydrogenase
NDRG1	N-myc downstream regulated 1
6-OHDA	6-hydroxydopamine
PHEO	pheochromocytoma
PGL	paraganglioma
PINK	PTEN-induced putative kinase
PPGL	pheochromocytoma/paraganglioma
ROS	reactive oxygen species
SDHB	succinate dehydrogenase B subunit
SOD	superoxide dismutase
VEGFA	vascular endothelial growth factor A
